# Detection of PDR5-mediated alachlor efflux using a chemically induced dimer biosensor

**DOI:** 10.1371/journal.pone.0334648

**Published:** 2025-10-27

**Authors:** Hadar Dubkin, Gil Zimran, Assaf Mosquna

**Affiliations:** The Robert H. Smith Institute of Plant Sciences and Genetics in Agriculture, the Hebrew University of Jerusalem, Rehovot, Israel; ICAR-Indian Agricultural Research Institute, INDIA

## Abstract

The development of herbicide-resistant crops is a key strategy for achieving efficient and sustainable agriculture. Herbicide tolerance can be conferred by transporters that mediate the efflux of herbicides, making them of particular interest for resistance development. However, identifying a transporter for a specific compound can be a challenging task. Here, we demonstrate the use of a synthetic, yeast two-hybrid-based molecular biosensor to identify transporter activity. A previously engineered biosensor responsive to the herbicide alachlor was utilized to monitor cytosolic alachlor levels in yeast, allowing for the evaluation of candidate transporters. A biosensor-reported shift in alachlor accumulation indicated the *Saccharomyces cerevisiae* transporter PDR5 as a potential mediator of alachlor efflux. PDR5’s effect on alachlor accumulation was suppressed by a known competing substrate of the transporter, validating its alachlor efflux activity. The ability of the biosensor yeast platform to report transporter activity and its inhibition via a fluorescence output underscores its potential as a tool for transporter-focused research.

## Introduction

Cellular detoxification mechanisms are defense strategies against toxic chemicals, observed across all kingdoms of life [1]. Ultimately, effective detoxification can confer tolerance to otherwise toxic xenobiotics. A core mechanism of cellular detoxification is facilitated by transmembrane protein transporters that mediate the active efflux of harmful compounds from the cytosol, thereby reducing the rate at which these compounds reach their intracellular targets [2,3]. In plants, it has been demonstrated that transporters can be utilized to generate herbicide resistance [3–7]. Accordingly, transporters capable of mediating herbicide cellular efflux are of particular interest for the development of herbicide-resistant crops.

A transporter capable of mediating the translocation of a compound of interest can be identified by its effect on the compound’s distribution across cellular compartments. However, tracking substance-specific trafficking is often challenging. As detoxification mechanisms operate at the single-cell level, they offer the advantage of being amenable to reconstitution in unicellular hosts, where intracellular and extracellular compartments are generally more accessible for measuring compound levels. Still, relying solely on conventional analytical methods for this purpose can be impractical for large-scale screening of transporter candidates. Recently, metabolomics-based assays have been developed to detect transport flux with reduced sample preparation and analytical complexity, for example, by utilizing isotope labeling [8]. High-throughput platforms can also be designed for compounds with visual properties or marked effects on host cell fitness [9–13]. Alternatively, targeted biosensing strategies may be applied. For example, certain phytohormones trigger chemically induced dimerization (CID) of cytosolic proteins [14]. Ligand-responsive protein complexes of this type can be adapted as sensors in living yeast cells by incorporating them into a yeast two-hybrid (Y2H) system [15,16]. In this configuration, intracellular ligand levels drive transcriptional activation of a reporter gene, enabling detection of ligand-related protein activity. In a study by Kanno et al., a native receptor complex of the phytohormone abscisic acid (ABA) was utilized to screen for an ABA transporter, successfully reflecting transporter-mediated ABA import into the yeast cytosol [17,18]. Notably, ABA receptor complexes incorporated into the Y2H system have also been employed for the development of biosensors targeting other small molecules. Several recent studies used this framework to engineer synthetic receptors, employing PYRABACTIN RESISTANCE 1-LIKE (PYL) proteins as scaffolds [19–22]. These efforts introduced novel biosensors for a variety of chemicals, including cannabinoids, insecticides, and herbicides, enabling their detection in living yeast cells.

The growing availability of molecular biosensors is advancing the efficient identification and characterization of proteins of interest [9,19–26]. In this work, we employed a synthetic receptor for alachlor, a preemergent herbicide from the chloroacetanilide family, to construct a platform for functional screening of transporter proteins in yeast with the aim of identifying potential herbicide exporters. Using this approach, we found that the *Saccharomyces cerevisiae* transporter PDR5 mediates the efflux of alachlor from the yeast cytosol.

## Results

### PDR5 expression reduces biosensor-reported alachlor levels in the yeast cytosol

To identify a transporter capable of mediating alachlor translocation, we began by evaluating a limited set of candidate transporters. Eight transporters from *Saccharomyces cerevisiae* and *Arabidopsis thaliana* were selected for testing for alachlor-related activity. Representatives of the ATP-binding cassette (ABC) transporter subfamily C, AtABCC1 and AtABCC2, were included due to their reported capacity to enhance plant tolerance to the herbicide disodium methanearsonate and the heavy metals cadmium and mercury [27,28]. Additionally, both proteins have been associated with the transport of metolachlor, a herbicide structurally related to alachlor [29,30]. Four ABCG subfamily transporters from yeast, *PDR5, SNQ2, PDR10* and *YOR1*, were also included as their expression was previously reported to be upregulated in yeast upon alachlor exposure [31].

An alachlor reporter yeast strain was constructed by genomic integration of a fluorescent reporter gene under GAL4 transcriptional regulation, as well as the Y2H system components *Brachypodium distachyon* PYL3 (BdPYL3) and PP2C44 (BdPP2C44), fused to the GAL4 activation and DNA-binding domains, respectively. Specifically, we used a synthetic alachlor-inducible BdPYL3 variant carrying the mutations V70I, A97C, H123N, L125A, F171C, and T172C[21]. Transporter genes were introduced into the reporter strain via a pESC shuttle vector. This design links alachlor-induced dimerization of the BdPYL3 variant and BdPP2C44 to transcriptional activation of a fluorescent reporter, enabling the monitoring of cytosolic alachlor levels and the potential detection of transporter-mediated alachlor translocation.

**Fig 1:**
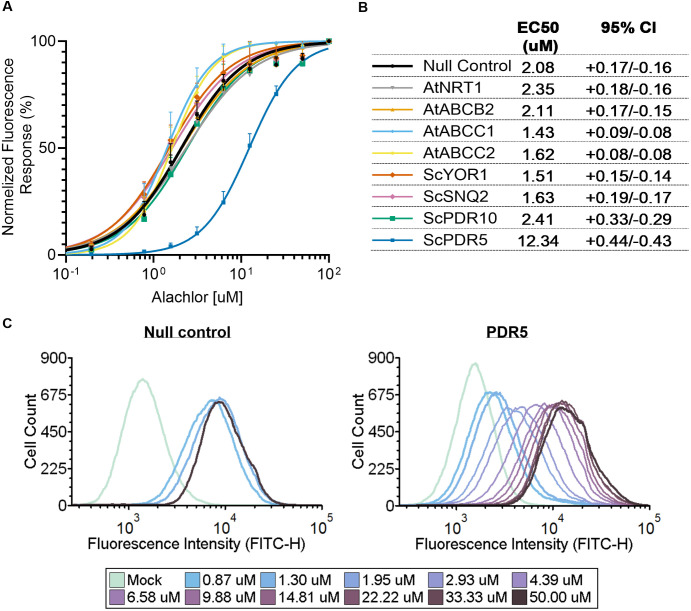
PDR5 expression reduces biosensor-reported alachlor levels in yeast cytosol. **A.** Concentration-dependent fluorescence responses of transporter-hosting yeast strains to alachlor. Normalized fluorescence response (%) was calculated as the ratio of mScarlet-I fluorescence (Ex/Em: 570/600 nm) to optical density (OD₆₀₀), normalized within each dataset using the lowest and highest values as estimates of basal and maximal responses, respectively. The x-axis indicates alachlor concentration in minimal media prior to incubation. Data points represent the mean of biological replicates (n = 4–5); error bars show 95% confidence intervals of the mean. Curves were fitted using a four-parameter logistic model with least squares fitting. Null control refers to the alachlor reporter strain carrying the pESC backbone lacking a transporter gene. **B.** Half-maximal effective concentration (EC₅₀) values derived from the fitted curves shown in panel A. 95% CI indicates the 95% confidence intervals of the EC₅₀ values. ’At’ and ’Sc’ prefixes indicate genes from *Arabidopsis thaliana* and *Saccharomyces cerevisiae*, respectively. AtNRT1, AtABCB2, AtABCC1 and AtABCC2 correspond to gene loci AT1G69850, AT4G25960, AT1G30400 and AT2G34660, respectively. **C.** PDR5’s impact on alachlor-induced fluorescence at the single-cell level. Histograms show the distributions of envyGFP fluorescence intensities across measured cells. Histogram color indicates the concentration of alachlor supplemented in the media prior to incubation. The left panel refers to the null control yeast; the right panel refers to the PDR5-overexpressing yeast. Different concentration sets were tested for each strain; shared concentrations included 0, 0.87, 1.30, and 50 µM. Gating details and derived response curves are provided in S1 Fig.

The alachlor concentration-dependent response of transporter-hosting yeast was assayed by measuring normalized mScarlet-I fluorescence emission upon exposure to increasing concentrations of alachlor. Among the tested transporters, only PDR5 substantially altered reported cytosolic alachlor levels, as indicated by a rightward shift in the fitted response curve (Fig 1A). Compared to the response exhibited by yeast transformed with the pESC backbone lacking a transporter gene (null control), the PDR5-overexpressing yeast displayed a distinctively and significantly higher half-maximal effective concentration (EC₅₀) (Fig 1B). The pronounced difference in alachlor concentration-dependent responses suggests that PDR5 activity reduces cytosolic alachlor levels when cells are exposed to a relatively broad concentration range, that is, relative to the sensitivity window provided by the reporter strain.

The fluorescence response of PDR5-overexpressing yeast to alachlor was further examined at the single-cell level using flow cytometry. Cells collected from cultures grown on alachlor-supplemented media were analyzed for their envyGFP fluorescence intensity. Median fluorescence intensities across alachlor concentrations were used to describe the concentration-dependent cellular response. Consistent with the divergence observed in whole-culture measurements, PDR5-overexpressing cells exhibited a higher EC₅₀ compared to the null control: 5.84 µM (95% confidence interval: 5.32–6.41 µM) for PDR5-overexpressing cells and 0.56 µM (95% confidence interval: 0.52–0.60 µM) for null control cells (response curves are shown in S1 Fig). These findings show that the impact of PDR5 expression on cytosolic alachlor levels, as reported by the biosensor, is evident at the single-cell level, supporting the notion that the transporter mediates alachlor efflux.

### Tacrolimus inhibits putative PDR5-mediated alachlor efflux

**Fig 2:**
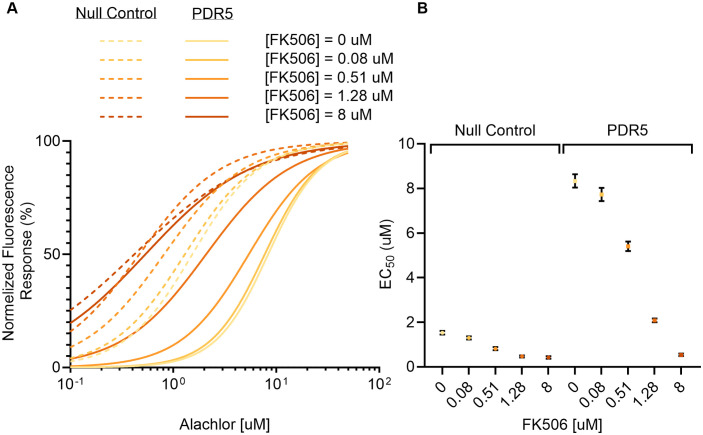
FK506 suppresses the PDR5-attributed differential fluorescence response to alachlor. **A.** Concentration-dependent fluorescence responses of PDR5-overexpressing and null control yeast strains to alachlor upon FK506 exposure. Normalized fluorescence response (%) was calculated as the ratio of mScarlet-I fluorescence (Ex/Em: 570/600 nm) to optical density (OD₆₀₀), normalized within each dataset using the lowest and highest values as estimates of basal and maximal responses, respectively. The x-axis indicates the alachlor concentration in minimal media prior to incubation. Curves were fitted using a four-parameter logistic model with least squares fitting based on six biological replicates per concentration (n = 6). Curve color corresponds to FK506 concentration. Dashed lines represent yeast carrying the pESC backbone without a transporter gene (null control); solid lines represent the PDR5-overexpressing yeast. **B.** Half-maximal effective concentration (EC₅₀) values derived from the response curves shown in panel **A.** Error bars indicate 95% confidence intervals. The left group corresponds to the null control yeast; the right group to the PDR5-overexpressing yeast.

To test whether PDR5 influences the intracellular response to alachlor via its efflux activity, we sought to modulate PDR5-mediated transport using one of its known substrates. Tacrolimus (FK506), a well-established substrate of PDR5, has previously been used to identify compounds associated with PDR5 activity [32–34]. We assayed yeast fluorescence following FK506 supplementation and found that FK506 suppressed the differential response to alachlor attributed to PDR5 activity. While FK506 alone did not stimulate fluorescence, its co-supplementation with alachlor shifted the alachlor-dependent fluorescence responses of both PDR5-overexpressing and null control yeast strains toward lower alachlor concentrations, indicating increased cytosolic availability of alachlor upon FK506 addition. However, the overall FK506-induced shift in alachlor EC₅₀ values was more pronounced in the PDR5-overexpressing yeast (Fig 2B). Importantly, as FK506 concentrations increased, the alachlor response curves of both strains gradually converged toward a similar provisional saturation limit (S2 Fig). Upon addition of approximately 1 µM FK506, the response of PDR5-overexpressing yeast became comparable to that of the FK506-untreated null control. These results indicate that additional FK506 target sites, namely, PDR5 transporter proteins, account for the disparity in alachlor responses between the PDR5-overexpressing and null control yeast strains. Thus, they support the interpretation that the biosensor-reported reduction in cytosolic alachlor accumulation in the PDR5-overexpressing yeast results from PDR5-mediated alachlor efflux.

## Discussion

In this study, we sought to identify a transporter capable of mediating the cellular efflux of the herbicide alachlor using a biosensor-based yeast platform. By evaluating the effects of candidate transporters on biosensor-reported cytosolic alachlor levels, we identified PDR5 as a potential alachlor exporter. The effect of PDR5 on cytosolic alachlor levels was suppressed upon the addition of a known competitive inhibitor of the transporter, supporting the conclusion that alachlor is a substrate of PDR5.

PDR5 is a well-characterized ABC transporter known for its role in yeast xenobiotic resistance, mediating the efflux of a broad range of compounds [35]. Its potential application in plants was previously explored by Remy et al., who showed that heterologous expression of PDR5 in *Arabidopsis thaliana* increased tolerance to several phytotoxins [36]. Combined with our findings in yeast, this highlights PDR5 as an attractive candidate for herbicide resistance development. Still, further work is required to establish the transporter’s utility. Assessing its activity toward alachlor in plants is needed to ensure that it is not conditional on processes tied to yeast physiology, such as specific chemical modifications. Moreover, transporter performance in planta may be marginal under physiologically relevant alachlor exposure levels and in the presence of a distinct set of competing substrates, particularly given PDR5’s promiscuity. Thus, its application may ultimately require refinement of transporter activity.

The assembly employed here, featuring the alachlor-inducible PYL variant, effectively reflected transporter activity via a fluorescence output. This result points to the potential of the modular yeast biosensor platform for transporter-focused research. Notably, given its proven track record in facilitating directed evolution workflows, the platform may also be utilized for the functional redesign of transporters [19–22]. For example, the experimental configuration used here to identify PDR5’s alachlor-related activity could be applied to screen for PDR5 variants with enhanced alachlor efflux capacity. PDR5 is a prime candidate for such manipulation, given its substrate promiscuity, the availability of detailed structural and functional data, and the ability of the alachlor reporter strain to visualize its activity at the single-cell level [35–42]. However, the usage of this platform for broad screening is susceptible to challenges common to other cell-based biosensor systems, and whether the current assembly can support robust enrichment of improved transporter variants remains to be determined. Nevertheless, given the abundance of recently developed synthetic PYL receptors, the prospect of a modular, fluorescence-based, high-throughput-compatible framework that facilitates the screening of transporters for activity toward a chemical of interest is compelling for diverse biotechnology endeavors, such as metabolic engineering and bioremediation [13,19–22,43,44].

Our work also underscores another utility of the yeast platform. We demonstrated that coupling a biosensor with a transporter via a shared substrate can enable the detection of transporter efflux inhibition. This suggests that the platform could be applied to identify transporter-chemical interactions. Overall, our findings highlight the platform as a versatile tool with broad applications in transporter-related research.

## Materials and methods

### Cloning

PCR amplification of DNA fragments was performed using either Phusion High-Fidelity DNA polymerase (New England Biolabs, MA, USA) or Rapid Taq DNA polymerase (Vazyme Biotech, Nanjing, PRC). Restriction enzymes were also sourced from New England Biolabs. Unless otherwise specified, multi-fragment assemblies were carried out using Gibson assembly [45].

### Reporter yeast strains

Yeast strains developed for this work were generated based on two previously constructed *Saccharomyces cerevisiae* strains: MaV99 + *GAL1/UASg:envyGFP:ADH1ter*@HO and Y190 + *GAL1/UASg:mScarlet-I:ADH1ter*@HO [16,21]. The Y2H-based alachlor biosensor components, BdPP2C44 co-receptor and BdPYL3 receptor variant (V70I, A97C, H123N, L125A, F171C, T172C), fused to the GAL4 DNA-binding and activation domains, respectively, were introduced into each of these strains. Expression cassettes were integrated into genomic loci ‘20’ and ‘21’, as named by Bai Flagfeldt et al., who identified them as high-expression sites [46]. Plasmids used for integration, p20StAD and p21StBD, were taken from a previous study [46]. Integration was achieved via homologous recombination, following yeast protocols [47]. Transformed colonies were screened by PCR using genomic DNA extracted by lithium acetate–SDS lysis, followed by ethanol precipitation [48]. Successful integration was confirmed by either Sanger sequencing or a functional fluorescence assay.

### Transporter-expression plasmid

A pESC shuttle vector was used for expression of candidate transporters in the alachlor reporter strains. The PGK1 promoter and CYC1 terminator, amplified from Y190 yeast genomic DNA, were used to drive transporter genes’ expression. Transporter coding sequences were amplified from either *Arabidopsis thaliana* cDNA or from *Saccharomyces cerevisiae* Y190 strain genomic DNA.

### Transporter activity assay via yeast fluorescence response

#### Plate reader.

Transporter-hosting yeast cultures’ concentration-dependent responses to alachlor were tested using a yeast two-hybrid (Y2H) assay in a 96-well plate format. Cultures (OD₆₀₀ = 0.05) in selective minimal media, supplemented with the relevant chemicals (alachlor or alachlor + FK506), were incubated for 22 hours at 30 °C and 1100 rpm on a Titramax shaker (Heidolph, DE). Following incubation, OD₆₀₀ and mScarlet-I fluorescence (excitation/emission: 570/605 nm) were measured using a Synergy H1 microplate reader (BioTek, VT, USA). Fluorescence values were divided by OD₆₀₀ to calculate relative fluorescence units (RFU). Samples exhibiting low optical density or signs of damage were excluded from analysis. For each yeast/chemical combination, 4–6 biological replicates were measured. RFU values were normalized independently within each replicate using the lowest and highest measured values, which served as estimates of basal and maximal responses, respectively. Dose-response curves were fitted to the pooled normalized data using a four-parameter logistic model with variable slope and least-squares fitting in GraphPad Prism (“Agonist vs. Normalized Response – Variable Slope”). All fitted curves reported here yielded R^^2^^ ≥ 0.95.

#### Flow cytometry.

Samples of the envyGFP reporter strain were analyzed using a BD Accuri C6 Plus flow cytometer (BD Biosciences, NJ). Cells from fresh cultures grown on selective minimal agar with varying alachlor concentrations (30 °C, 3 days) were harvested, resuspended in ice-cold PBS, and kept on ice prior to analysis to arrest growth. Forward scatter area (FSC-A) vs. height (FSC-H) plots were used to gate the main cluster of putative singlet cells (FSC-A/FSC-H ≈ 1), obtaining a minimum of 50,000 events per sample (S1 Fig). Histograms of envY-GFP fluorescence intensities from gated cell populations were displayed in FCS Express 7 using the default resolution of 1024 bins. Median fluorescence intensities were used to quantify the concentration-dependent responses to alachlor. Normalization and curve fitting were performed as described above.

## Supporting information

S1 Fig PDR5’s impact on biosensor-reported alachlor levels at the single-cell level. Yeast single-cell envyGFP fluorescence responses to alachlor, measured by flow cytometry. **A, B:** Histograms showing the distributions of envyGFP fluorescence intensity for gated cell events (gating shown in panel C). Histogram color indicates the alachlor concentration in the media prior to incubation. **A.** Null control yeast. **B.** PDR5-overexpressing yeast. Different sets of concentrations were tested for each yeast strain; shared concentrations include 0, 0.87, 1.30, and 50 µM. **C.** Gating applied to isolate the main cluster of singlet cells, shown on a representative sample. **D.** Concentration-dependent responses of yeast cells to alachlor. Response curves were generated from the median fluorescence intensities of the distributions shown in panels A and B. Median values were normalized within each dataset using the lowest and highest values as estimates of basal and maximal responses, respectively. Curves were fitted to the normalized data using a four-parameter logistic model with least squares fitting.
(PDF)

S2 Fig FK506 suppresses the PDR5-attributed differential fluorescence response to alachlor. Concentration-dependent fluorescence responses of PDR5-overexpressing and null control yeast strains to alachlor upon FK506 exposure. Normalized fluorescence response (%) was calculated as the ratio of mScarlet-I fluorescence (Ex/Em: 570/600 nm) to optical density (OD₆₀₀), normalized within each dataset using the lowest and highest values as estimates of basal and maximal responses, respectively. The x-axis indicates the alachlor concentration in minimal media prior to incubation. Curves were fitted using a four-parameter logistic model with least squares fitting based on six biological replicates per concentration (n = 6). Curve color corresponds to FK506 concentration. Dashed lines represent the null control yeast carrying the pESC backbone without a transporter gene; solid lines represent the PDR5-overexpressing yeast.
(PDF)
