# Supplementary material for: Detection of PDR5-mediated alachlor efflux using a chemically induced dimer biosensor
Source: PLoS One. 2025 Oct 27;20(10):e0334648. doi: 10.1371/journal.pone.0334648 (PMC12558554; doi:10.1371/journal.pone.0334648)
Supplement: S1 Fig — Yeast single-cell envyGFP fluorescence responses to alachlor, measured by flow cytometry. A, B: Histograms showing the distributions of envyGFP fluorescence intensity for gated cell events (gating shown in panel C). Histogram color indicates the alachlor concentration in the media prior to incubation. A. Null control yeast. B. PDR5-overexpressing yeast. Different sets of concentrations were tested for each yeast strain; shared concentrations include 0, 0.87, 1.30, and 50 µM. C. Gating applied to isolate the main cluster of singlet cells, shown on a representative sample. D. Concentration-dependent responses of yeast cells to alachlor. Response curves were generated from the median fluorescence intensities of the distributions shown in panels A and B. Median values were normalized within each dataset using the lowest and highest values as estimates of basal and maximal responses, respectively. Curves were fitted to the normalized data using a four-parameter logistic model with least squares fitting. (PDF) [file pone.0334648.s001.pdf]

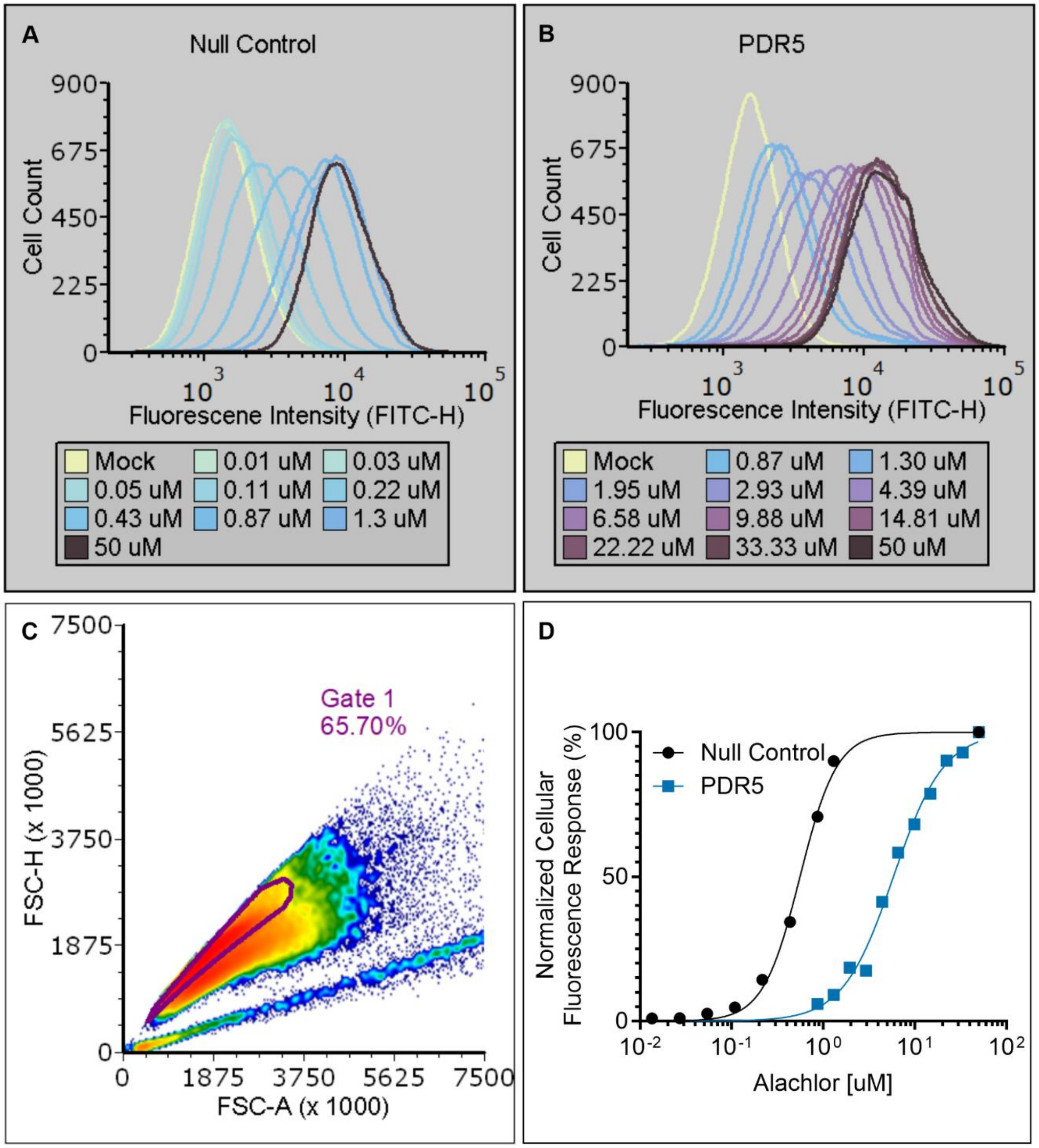

S1 Figure. PDR5's impact on biosensor-reported alachlor levels at the single-cell level. Yeast single-cell envyGFP fluorescence responses to alachlor, measured by flow cytometry. **A, B:** Histograms showing the distributions of envyGFP fluorescence intensity for gated cell events (gating shown in panel C). Histogram color indicates the alachlor concentration in the media prior to incubation. **A.** Null control yeast. **B.** PDR5-overexpressing yeast. Different sets of concentrations were tested for each yeast strain; shared concentrations include 0, 0.86, 1.30, and 50 μM. **C.** Gating applied

to isolate the main cluster of singlet cells, shown on a representative sample. **D.** Concentration-dependent responses of yeast cells to alachlor. Response curves were generated from the median fluorescence intensities of the distributions shown in panels A and B. Median values were normalized within each dataset using the lowest and highest values as estimates of basal and maximal responses, respectively. Curves were fitted to the normalized data using a four-parameter logistic model with least squares fitting.
