# Supplementary material for: Detection of PDR5-mediated alachlor efflux using a chemically induced dimer biosensor
Source: PLoS One. 2025 Oct 27;20(10):e0334648. doi: 10.1371/journal.pone.0334648 (PMC12558554; doi:10.1371/journal.pone.0334648)
Supplement: S2 Fig — Concentration-dependent fluorescence responses of PDR5-overexpressing and null control yeast strains to alachlor upon FK506 exposure. Normalized fluorescence response (%) was calculated as the ratio of mScarlet-I fluorescence (Ex/Em: 570/600 nm) to optical density (OD₆₀₀), normalized within each dataset using the lowest and highest values as estimates of basal and maximal responses, respectively. The x-axis indicates the alachlor concentration in minimal media prior to incubation. Curves were fitted using a four-parameter logistic model with least squares fitting based on six biological replicates per concentration (n = 6). Curve color corresponds to FK506 concentration. Dashed lines represent the null control yeast carrying the pESC backbone without a transporter gene; solid lines represent the PDR5-overexpressing yeast. (PDF) [file pone.0334648.s002.pdf]

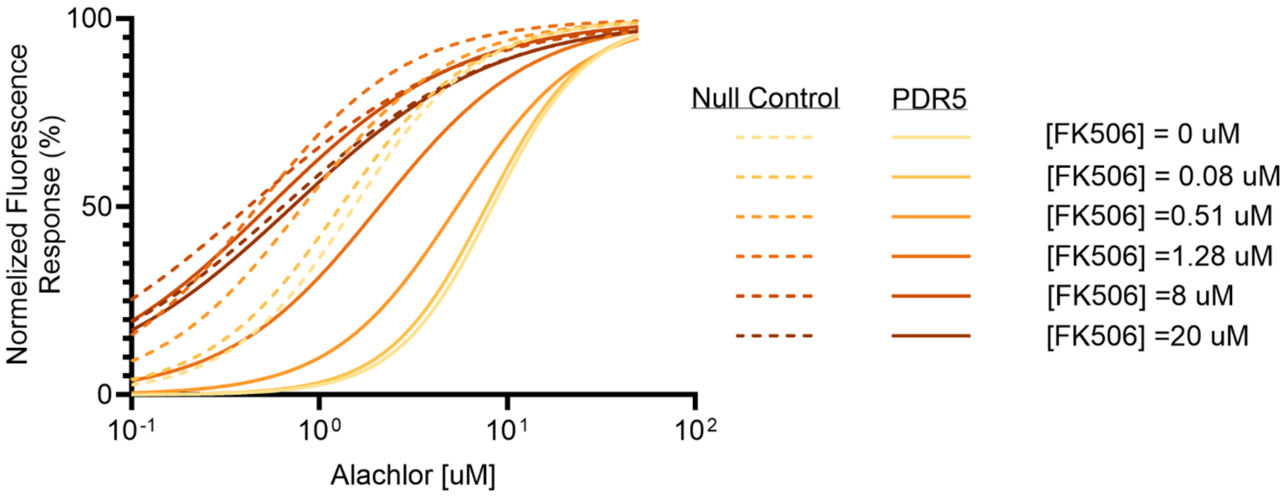

S2 Figure. FK506 suppresses the PDR5-attributed differential fluorescence response to alachlor.

Concentration-dependent fluorescence responses of PDR5-overexpressing and null control yeast strains to alachlor upon FK506 exposure. Normalized fluorescence response (%) was calculated as the ratio of mScarlet-I fluorescence (Ex/Em: 570/600 nm) to optical density (OD_{600}), normalized within each dataset using the lowest and highest values as estimates of basal and maximal responses, respectively. The x-axis indicates the alachlor concentration in minimal media prior to incubation. Curves were fitted using a four-parameter logistic model with least squares fitting based on six biological replicates per concentration (n = 6). Curve color corresponds to FK506 concentration. Dashed lines represent the null control yeast carrying the pESC backbone without a transporter gene; solid lines represent the PDR5-overexpressing yeast.
